# Two *Streptococcus agalactiae* ST283 invasive infections in Portugal raise the possibility of the emergence of this unusual lineage in Europe

**DOI:** 10.3389/fmicb.2025.1712362

**Published:** 2026-01-26

**Authors:** Elisabete R. Martins, Mykyta Forofontov, José Mota Freitas, José Melo-Cristino, Mário Ramirez, Portuguese Group for the Study of Streptococcal Infections, Miguel Seruca, João Marques, Isabel Peres, Teresa Pina, Isabel Lourenço, Cristina Marcelo, Isabel Daniel, Odete Chantre, Vasco Mendes, Margarida Pinto, Rui Ferreira, Marília Gião, Teresa Vaz, Catarina Chaves, Rui Tomé Ribeiro, Celeste Pontes, Luísa Boaventura, Teresa Reis, Henrique Oliveira, Ana Aguiar, Mariana Silva, Hugo Loureiro, Adriana Pedrosa, Hermínia Costa, Maria Fátima Silva, Maria Amélia Afonso, Mariana Fardilha, Natália Novais, Isabel Brito, Ana Bruschy Fonseca, Luís Marques Lito, Maria Ana Pessanha, Elsa Gonçalves, Teresa Morais, Cristina Toscano, Elisabete Cristovam, Filomena Martins, Paulo Lopes, Angelina Lameirão, Gabriela Abreu, Aurélia Selaru, Ana Paula Mota Vieira, Margarida Tomaz, Marta Nicolau, Cláudia Ferreira, Ana Paula Castro, Helena Ramos, Virgínia Lopes, Hugo Cruz, Nádia Martins, Carla Leite, Alexandra Estrada, Filipa Bazenga, Fernando Fonseca, Ana Paula Castro, Filipa Vicente, Margarida Pereira, Nuno Canhoto, Teresa Afonso, Maria Paula Falcão, Ilse Fontes, Rui Semedo, Gina Marrão, Filipa Silva, Manuela Ribeiro, Helena Gonçalves, Alberta Faustino, Maria Cármen Iglesias, Adriana Coutinho, Ana Bela Correia, Maria Luísa Gonçalves, Elzara Aliyeva, Sandra Schäfer, Clara Portugal, Isabel Monge, Luísa Sancho, José Diogo, Filipa Fortunato, Leonardo Carneiro, José Marta, Isabel Nascimento, Nadiya Kruptsala, Cláudia Fidalgo, Raquel Diaz, Sónia Ferreira, Inês Cravo Roxo, Elmano Ramalheira, Isabel Vale, Maria João Tomás, José Miguel Ribeiro, Maria Antónia Read, Valquíria Alves, Margarida Monteiro, Dr. João Faria, Margarida Rodrigues, Sandra Vieira, Maria Favila Menezes, Elsa Calado, Bruno Miguel, L. Nogueira Martins, Maria José Rego de Sousa, Paula Pinto, José Germano de Sousa, Ana Custódio, Maria Calle, Mariana Bettencourt Viana, Marvin Oliveira, Hugo Macedo, Svetlana Zhelezovskaya, Isaura Terra, Vitória Rodrigues, Sofia Marques, Joana Selada, Patrícia Pereira, Manuela Azevedo, Jesuína Duarte, Joana Bernardo, Inês Tapadinhas, Ana Filipa Resende, Andreia Bernardo, Luísa Oliveira, Susana Banza, Ezequiel Moreira, Carla Ferreira, Adília Vicente, Cristina Bragança, Maria Lucas, Paula Gouveia Pestana, Patrícia Amantegui, Cristina Mota Preto, Sara F. Sampaio, Fátima Vale, Joana Ramos, Rita Gralha, Luís Fialho, Paula Gama, Ana Helena Correia, Paula Pinto, Ana Jesus, Marisol Lourinha

**Affiliations:** 1 GIMM - Gulbenkian Institute for Molecular Medicine, Lisbon, Portugal; 2 Instituto de Microbiologia, Faculdade de Medicina, Universidade de Lisboa, Lisbon, Portugal; 3 Unidade Local de Saúde do Alto Minho, Viana do Castelo, Portugal

**Keywords:** epidemiological surveillance, group B streptococcus, invasive infections, ST283, foodborne, *Streptococcus agalactiae*, whole-genome sequencing

## Abstract

The importance of invasive *Streptococcus agalactiae* (Lancefield group B streptococci – GBS) disease among adults is increasingly recognized worldwide. Acquisition from other humans or an individual’s own microbiota is thought to be the origin of the diverse GBS lineages causing these infections. We report two cases in three consecutive years (2016–2018) of invasive GBS disease in Portugal due to the unusual ST283 lineage, a hypervirulent clone circulating in Southeast Asia (SEA) and associated to foodborne transmission and large disease outbreaks among previously healthy younger adults. The need for genetic analyses, which are infrequently performed, to identify ST283 may mean that cases of ST283 occur sporadically outside SEA but go undetected.

## 1 Introduction

Most *Streptococcus agalactiae* or Lancefield group B streptococci (GBS) infections are believed to be endogenous or result from transmission from other humans (Martins et al., 2022). A large foodborne outbreak of invasive GBS disease (iGBS) in Singapore in 2015 was caused by an unusual, hypervirulent zoonotic lineage identified by traditional seven-gene multilocus sequence typing (MLST) as sequence type 283 (ST283), which was associated with the consumption of raw freshwater fish (Kalimuddin et al., 2017). The Singapore outbreak was remarkable for involving numerous cases of invasive disease among previously healthy adults, suggesting that this clone possesses enhanced virulence when compared to typical iGBS lineages. Subsequent studies showed that ST283 has been a frequent cause of GBS infections in both humans and freshwater fish across Southeast Asia (SEA) for at least two decades (Barkham et al., 2018, 2023; Zohari et al., 2023). This growing body of evidence led FAO to issue a risk assessment of ST283 in the region (FAO, 2021). Human invasive infections have been reported in Thailand, Laos, Vietnam, Hong Kong, and China, often associated with consumption of raw freshwater fish (Barkham et al., 2019; Aiewsakun et al., 2022; Luangraj et al., 2022). In parallel, ST283 has caused recurrent streptococcosis outbreaks in farmed tilapia across Thailand, Vietnam, Malaysia, and Singapore (Delannoy et al., 2013; Barkham et al., 2018; FAO, 2021), highlighting its long-standing regional circulation.

Disease by ST283 occurred in younger patients, with less comorbidities and presented more frequently as meningoencephalitis, native joint infection and spinal infection than those caused by other GBS lineages (Kalimuddin et al., 2017). Epidemiology, case–control data, and whole-genome comparisons linked human cases to consumption of raw freshwater fish; and genomic analyses showed human and fish ST283 isolates cluster tightly, consistent with a shared reservoir or cross-species transmission (Rajendram et al., 2016; Barkham et al., 2019). The global reach of current food chains raises the possibility of transmission of ST283 outside of SEA through contaminated fish. There is already a report in the literature of diseased fish in Brazil (Leal et al., 2019), highlighting the potential global spread of this lineage, but there is limited data on human infections outside SEA, with only five cases reported to date.

Here we describe two cases of invasive GBS disease caused by ST283 in Portugal identified within a long-term national surveillance effort of invasive GBS infections. To contextualize these rare cases within the global epidemiology of this lineage we genomically characterize our isolates. Our report documents further ST283 infections, indicating that ST283 may occur beyond SEA suggesting the ST283 lineage may pose additional challenges to the management of GBS infections worldwide due to its virulence and potential transmission through the food chain.

## 2 Materials and methods

### 2.1 Surveillance of GBS invasive infections (iGBS) among non-pregnant adults in Portugal

We conduct an ongoing surveillance of iGBS among non-pregnant adults (≥18 years) in Portugal. Microbiology laboratories in Portuguese hospitals participating in our surveillance network are asked to submit to a central laboratory GBS isolates recovered from normally sterile sites. In the period 2016–2021 a total of 585 unique isolates of iGBS were submitted for characterization. Capsular serotyping (Ia, Ib, II-IX) was done initially by the slide agglutination IMMULEX STREP-B Kit (Statens Serum Institute, Copenhagen, Denmark) and later by genomic analysis (see below). Antimicrobial susceptibility testing was performed by disk diffusion following the EUCAST methods and interpretative criteria (EUCAST, 2021). All isolates were tested for susceptibility to penicillin G, erythromycin, clindamycin, vancomycin, chloramphenicol, norfloxacin, gentamicin, streptomycin, and tetracycline. A disk diffusion screening test for high-level aminoglycoside resistance (HLAR) was also performed according to the EUCAST methods and interpretative criteria for *Enterococcus* species (EUCAST, 2021).

While our network comprised most hospital microbiology laboratories in Portugal, participation was voluntary and the surveillance system is therefore not population-based. No audits were performed to assess reporting completeness, and it is possible that not all laboratory-confirmed cases of iGBS disease occurring within the network were reported. Only the isolate, isolation date, source of isolation, patient age and gender were provided, so the data is irretrievably unlinked from an identifiable person. These activities were considered surveillance activities exempt from ethical approval and from individual informed consent under applicable national data-protection and public-health frameworks.

### 2.2 Preparation of DNA for sequencing and high-throughput sequencing

Genomic DNA was extracted from cultures of GBS grown overnight in Todd-Hewitt broth (Oxoid, Basingstoke, UK) using the PureLink Genomic DNA Mini Kit (Invitrogen, Carlsbad, CA, USA), according to the manufacturer’s instructions. The initial bacterial lysis step was carried out in the presence of 15U of mutanolysin (Sigma-Aldrich, St. Louis, MO, USA). Whole-genome sequencing libraries were generated using the Nextera DNA library preparation kit (Illumina, San Diego, CA, USA). The libraries were sequenced in an Illumina NextSeq instrument and sequencing data of our three isolates is available in the European Nucleotide Archive, ENA; accession number PRJNA1000441. Reads were assembled using the INNUca pipeline with default parameters and with -s *S. agalactiae* -g 2.1.^1^

#### 2.2.1 Data retrieval

To identify genomes of ST283 isolates we searched PubMed for papers that referenced *S. agalactiae* ST283 (December 2023) and retrieved all the data associated with those papers. In those cases where only reads were available, these were assembled using the same methods described above.

#### 2.2.2 Identification of ST283 samples

To identify the samples of interest, we performed *in silico* ST prediction using MLST v2.19.0^2^ with default parameters and the database updated in December 2023. Those assemblies that did not have sequence type 283 (ST283) or were too fragmented (>200 contigs) were removed from further analysis.

### 2.3 Creation of a whole-genome multilocus sequence typing (wgMLST)

To establish relationships between our isolates and those identified in other studies we created a whole-genome multilocus sequence typing (wgMLST) schema using all available GBS ST283 genomic samples and chewBBACA version 3.2.0 (Silva et al., 2018). The schema is deposited in Zenodo^3^ (Forofontov and Ramirez, 2024). Samples with an unusually low number of loci detected upon wgMLST analysis or that lacked one of the variants of the PI-2 locus (see below) were excluded from the final analysis. The list of genomes used in the analysis is deposited in Zenodo (see text footnote 3) (Forofontov and Ramirez, 2024).

#### 2.3.1 wgMLST analysis of ST283 isolates

Allelic profiles of the core loci [shared by 100% of the isolates under analysis (cgMLST_100_)] were used to create a minimum spanning tree-like (MST) representation with the online version of PHYLOViZ (Ribeiro-Gonçalves et al., 2016). We aimed to verify the relationships between isolates and place our isolates within the context of the known variability of ST283.

### 2.4 Detection of antimicrobial resistance and virulence genes

To detect antimicrobial resistance and virulence factor encoding genes we employed Abricate version 1.0.1 with default parameters^4^ and the ncbi and vfdb (Liu et al., 2021) databases updated in December 2023. The AMR gene screen included macrolide- and lincosamide-resistance genes *ermB* and *mefA*/*E*, tetracycline-resistance genes *tetM* and *tetO*, and aminoglycoside-resistance genes such as *ant*(6)-Ia and *aph*(3’)-III. Virulence gene screening encompassed the capsular polysaccharide (*cps*) operon, pili islands PI-1, PI-2a, and PI-2b, the alpha-like protein family (alpha, *rib*, *alp*2/3, *eps*), the cyl operon including *cylE*, and additional streptococcal virulence loci such as *hylB*, *scpB*, and *lmb*. Additionally, the serotype, surface antigen encoding genes and pilus island loci were detected with Seq_typing^5^ and the various –org gbs options. The *parC* and *gyrB* alleles were identified in the wgMLST schema and were analyzed manually for the presence of relevant mutations in the quinolone resistance determining regions using Geneious 8.1.9.

## 3 Results

Retrospectively, we found three isolates presenting ST283, corresponding to 2 out of 585 iGBS cases (0.34%) in this period (data available in the European Nucleotide Archive, accession number PRJNA1000441).

One isolate was recovered in 2016 from blood of a patient with septic arthritis in a hospital in the north of Portugal. Two isolates were recovered in 2018 from a patient (blood and CSF, respectively) with meningitis in a Lisbon hospital. Both patients were males, non-immigrant Portuguese citizens, over 70 years old. The meningitis patient died during the hospital stay. While arthritis and meningitis are frequent presentations of ST283 infections in SEA, ST283 patients tended to be younger in SEA (Kalimuddin et al., 2017). However, in the recent outbreak in Hong Kong, in which foodborne transmission was considered unlikely, the age of the patients with ST283 cases was not different from that of other iGBS cases (Li et al., 2025). We had no access to additional information on the patients’ history or clinical details of the episodes, but the chronology (2 years apart) and geography (>300 km distance) suggested that the two cases were not related to each other. All isolates were susceptible to penicillin, erythromycin, clindamycin, chloramphenicol, vancomycin, streptomycin, and gentamicin; and resistant to tetracycline and norfloxacin, following the EUCAST methods and interpretative criteria (EUCAST, 2021).

Draft genomic information of the three isolates was used to compare them to other ST283 deposited in public databases. Allelic profiles of core-genome multilocus sequence typing [loci shared by 100% of the isolates under analysis (cgMLST_100_)] were used to create a minimum-spanning tree-like (MST) representation (Ribeiro-Gonçalves et al., 2016; Silva et al., 2018). In agreement with the phenotypic antimicrobial susceptibility, all three Portuguese isolates carried the *tetM* gene and the same mutations within the quinolone resistance determining regions of *gyrA* (S81L) and *par*C (S79Y) (Table 1), previously described as associated to reduced quinolone susceptibility in GBS (Kawamura et al., 2003; Nagano et al., 2012). Genomic analysis also confirmed the presence of genes encoding the alpha C surface protein (*bca*), pilus islands PI-1 and PI-2a, and the type III capsular polysaccharide locus (Table 1), as is characteristic of ST283.

**TABLE 1:** Distribution of serotype, antimicrobial resistance profiles and major virulence factors detected among ST283 iGBS isolates recovered in Portugal.

Capsular type	Antimicrobial resistance		Virulence factors (gene)
	Disk diffusion^a^	Genetic determinants	
III	TET NOR	*tetM* *gyrA* (S81L), *parC* (S79Y)	CPS operon (*cps*, *neu*) Pilus islands PI-1, PI-2a (*pilA*, *pilC*, *srtC1*-*srtC4*) surface protein alpha (*bca*) Adhesin of Collagen-like Protein C (*acpC*) β-hemolysin/cytolysin (*cyl* genes) Hyaluronidase (*hylB*) CAMP factor (*cfa*/*cfb*)

^a^TET, tetracycline; NOR, norfloxacin.

**FIGURE 1:**
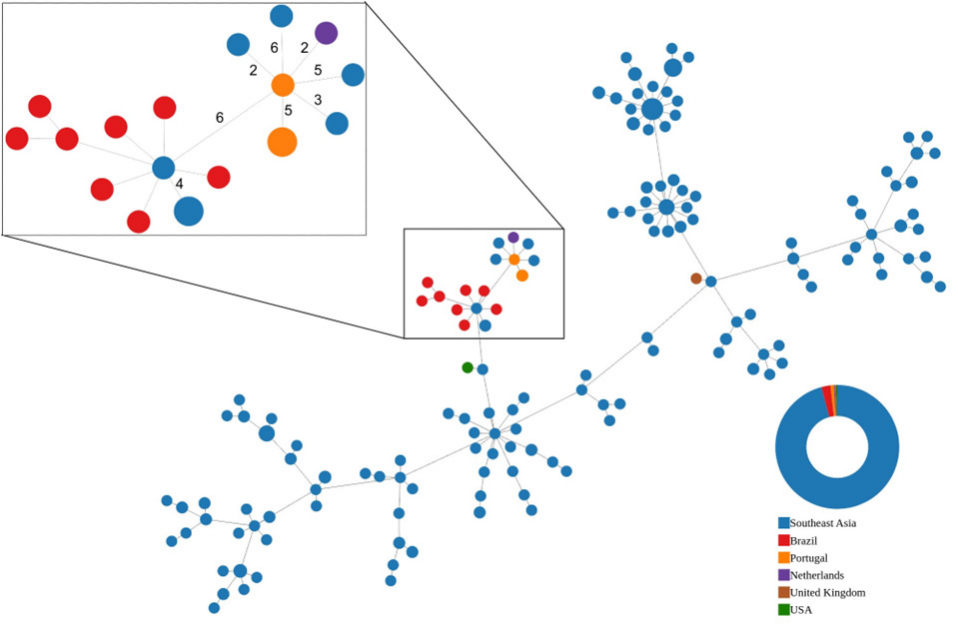
Minimum-spanning tree-like (MST) visualization of the allelic differences of publicly available ST283 core genomes. Nodes represent strains. Node size is proportional to the number of strains sharing the same cgMLST_100_ profile and are colored according to their region/country of origin. Southeast Asia (SEA) region includes the following countries/cities: Singapore, Malaysia, Laos, Thailand, Hong Kong, and Vietnam. SEA isolates were recovered from both human and fish. Isolates from Brazil were recovered from fish only. Single isolates from the Netherlands, United Kingdom and the United States were isolated from human infections. The cgMLST_100_ of all represented strains is comprised of 1,530 loci, while the subset highlighted in the inset has a cgMLST_100_ comprised of 1,696 loci. Edges link the closest pairs of strains. In the inset, edges are labeled to indicate the number of allelic differences if these are <10. All strains represented in the inset lack the *scp*B and *lmb* genes and all carry the same mutations within the QRDR regions of *gyr*A (S81L) and *par*C (S79Y), previously described as associated to reduced quinolone susceptibility in GBS (all ST283 isolates represented carry the same *gyr*A allele encoding the S81L mutation).

Overall, the cgMLST_100_ revealed limited variation between ST283 isolates (maximum difference of 45 alleles among 1,530 shared core loci, or 2.9% difference) (Figure 1), as reported previously (Schar et al., 2023). The genomic information also does not support an epidemiological link between the two Portuguese cases, with the isolates recovered from blood and CSF of the same patient showing no allelic differences but differing from the isolate of the other case in five loci (cgMLST_100_ of 1,696 loci, 0.3% difference). Although these isolates grouped together in the MST, they had similar or even higher number of allelic differences to geographically and temporally unrelated isolates (Figure 1).

Only five ST283 isolates of human origin have been reported outside SEA: two from France (no genomic information available), one from the UK, one from the Netherlands (Barkham et al., 2019), and one from the USA (McGee et al., 2021; Figure 1). The Portuguese isolates grouped close to the isolate recovered in the Netherlands but with a similar allelic difference to that of isolates recovered in different countries of SEA. Interestingly, the Portuguese isolates and its closest neighbors in the MST, lacked the *scpB* and *lmb* genes, which have been associated to virulence in human infections but that are postulated to have been frequently lost during ST283 evolution (Schar et al., 2023) and shared the same mutations in *gyrA* and *parC*. Similarly a sub-cluster of isolates in the recent Hong Kong outbreak also lacked the *scpB* and *lmb* genes (Li et al., 2025).

## 4 Discussion

Although ST283 has been associated with fish and human infections, recently ST283 was also detected in fecal carriage in 2.7% of a convenience sample of the population in northeast Thailand (Barkham et al., 2023). The dynamics of human carriage of ST283 and its contribution to human-to-human transmission, human disease, and the contamination of aquaculture, remain uncertain but the authors propose that human carriers could have been the original source of ST283 and contribute to human-to-human transmission (Barkham et al., 2023), as suggested for other lineages of GBS (Martins et al., 2022). A genomic study also determined that human-to-fish transmission was more frequent than in the reverse direction, but argued that fish could still constitute an important reservoir for ST283 (Schar et al., 2023). The importance of contaminated fish consumption in the 2015–2016 outbreak in Singapore, supported by subsequent genomic studies, confirmed that iGBS ST283 infection can be acquired through the food chain (FAO, 2021). Detection of ST283 in diseased farmed fish in Brazil led to fears of similar outbreaks in South America (Leal et al., 2019) but no human cases were subsequently reported, although the lack of genetic analysis may mean that any cases may have gone undetected. Aquaculture activity in Portugal has focused essentially on the production of marine species with freshwater aquaculture representing <5% of the total aquaculture production (Rocha et al., 2022), so it seems unlikely that these patients acquired the infection from fish farmed in Portugal, although we cannot exclude a role of imported fresh or frozen fish. The traditional Portuguese diet primarily features fully cooked or cured fish dishes; however, globalization has led to a rise in the popularity of raw fish preparations, such as sushi, sashimi, and ceviche.

Given the short time documented between exposure and development of disease in ST283 infections (Luangraj et al., 2022), and although we have no definite information on previous recent travel of the patients to SEA, it is unlikely that these infections were acquired in SEA, although this cannot be completely excluded.

The detection of fluoroquinolone resistance–associated QRDR mutations in our isolates is noteworthy in the context of ST283 epidemiology. Substitutions such as *gyrA* S81L and *parC* S79Y are well-established determinants of high-level fluoroquinolone resistance in GBS, having been described in resistant human isolates from multiple lineages (Ouancharee et al., 2025) and in enrofloxacin-resistant fish-derived strains from freshwater aquaculture settings (Lukkana et al., 2016). These mutations are present in our isolates as well as other isolates recovered from humans in SEA and The Netherlands (Figure 1). Given the widespread use of enrofloxacin in freshwater aquaculture in SEA and the established role of raw freshwater fish consumption in ST283 transmission, these findings highlight the potential for crosssector selection, particularly as fluoroquinolones are rarely used to treat human GBS infections and underscore the importance of sustained genomic surveillance to detect and monitor resistant ST283 lineages.

In addition, the absence of *scpB* and *lmb* in our isolates and their closest neighbors in the MST aligns with broader ST283 evolutionary patterns. Although these adhesins are commonly associated with human GBS virulence, several genomic studies have shown that their loss is frequent across ST283 lineages, including in fish-derived isolates outside Southeast Asia (Leal et al., 2019; Schar et al., 2023; Li et al., 2025). This suggests that deletion of *scpB* and *lmb* is a stable feature of ST283 evolution rather than an adaptation restricted to human infection. Accordingly, their absence in our isolates does not exclude an animal or foodborne source and is consistent with the genome architecture observed across geographically and ecologically diverse ST283 reservoirs.

Our study has limitations. Firstly, we had no access to detailed clinical information for the two patients, limiting our ability to assess potential risk factors, underlying conditions, or travel history that might have contributed to the infection by ST283. Secondly, the number of ST283 isolates analyzed is small, which constrains epidemiological inferences based on genomic information. Thirdly, our genomic analysis is limited by the availability and completeness of publicly accessible ST283 genomes from SEA and other regions, which may influence comparative interpretations. Finally, although we discuss possible epidemiological scenarios, our study design does not allow us to establish direct links between human cases, food sources, or environmental reservoirs.

We have kept a surveillance of iGBS in Portugal since 2001 and have ST information for 1,697 isolates. None of the isolates before the current study presented ST283. The identification of two ST283 iGBS cases in a 3-year period (2016–2018) was worrying but no ST283 isolates causing iGBS were recovered in subsequent years (2019–2021), suggesting there was no sustained risk. Although the ultimate source of the isolates remains unknown, taking together the previous episodic cases of ST283 iGBS in Europe and the genomic information available from European isolates, this is consistent with occasional, independent introductions of ST283 into Europe rather than the circulation of ST283 within Europe. The necessity of determining the ST for identification of ST283 means that cases may go undetected if MLST or genomic analyses are not performed. Given the possibility of ST283 transmission through the food chain and the prospect of asymptomatic gastrointestinal colonization, reinforcing iGBS vigilance is warranted to identify further cases and inform measures to prevent the potential dissemination of this enhanced virulence GBS lineage outside of SEA.

## Members of the Portuguese Group for the Study of Streptococcal Infections

Miguel Seruca, João Marques, Isabel Peres, Teresa Pina, Isabel Lourenço, Cristina Marcelo, Isabel Daniel, Odete Chantre, Vasco Mendes, Margarida Pinto (Unidade Local de Saúde São José, Lisboa, Portugal); Rui Ferreira, Marília Gião, Teresa Vaz (Unidade Local de Saúde Algarve, Faro e Portimão, Portugal); Catarina Chaves, Rui Tomé Ribeiro, Celeste Pontes, Luísa Boaventura, Teresa Reis, Henrique Oliveira (Unidade Local de Saúde Coimbra, Coimbra, Portugal); Ana Aguiar, Mariana Silva, Hugo Loureiro, Adriana Pedrosa, Hermínia Costa, Maria Fátima Silva, Maria Amélia Afonso (Unidade Local de Saúde Entre Douro e Vouga, Santa Maria da Feira, Portugal); Mariana Fardilha, Natália Novais, Isabel Brito (Unidade Local de Saúde Baixo Mondego, Figueira da Foz, Portugal); Ana Bruschy Fonseca, Luís Marques Lito (Unidade Local de Saúde Santa Maria, Lisboa, Portugal); Maria Ana Pessanha, Elsa Gonçalves, Teresa Morais, Cristina Toscano, Elisabete Cristovam, Filomena Martins (Unidade Local de Saúde Lisboa Ocidental, Lisboa, Portugal); Paulo Lopes, Angelina Lameirão, Gabriela Abreu, Aurélia Selaru (Unidade Local de Saúde Vila Nova de Gaia/Espinho, Vila Nova de Gaia e Espinho, Portugal); Ana Paula Mota Vieira, Margarida Tomaz (Unidade Local de Saúde Alto Ave, Guimarães, Portugal); Marta Nicolau, Cláudia Ferreira (Unidade Local de Saúde Baixo Alentejo, Beja, Portugal); Ana Paula Castro, Helena Ramos, Virgínia Lopes, Hugo Cruz (Unidade Local de Saúde Santo António, Porto, Portugal); Nádia Martins, Carla Leite, Alexandra Estrada, Filipa Bazenga, Fernando Fonseca (Unidade Local de Saúde Póvoa do Varzim/Vila do Conde, Póvoa do Varzim e Vila do Conde, Portugal); Ana Paula Castro (Unidade Local de Saúde Trás-os-Montes e Alto Douro, Vila Real, Peso da Régua e Chaves, Portugal); Filipa Vicente, Margarida Pereira, Nuno Canhoto, Teresa Afonso (Serviço de Saúde da Região Autónoma da Madeira, Funchal, Portugal); Maria Paula Falcão, Ilse Fontes, Rui Semedo (Unidade Local de Saúde Alto Alentejo – Elvas e Portalegre, Portugal); Gina Marrão, Filipa Silva (Unidade Local de Saúde Região de Leiria, Portugal); Manuela Ribeiro, Helena Gonçalves (Unidade Local de Saúde São João, Porto, Portugal); Alberta Faustino, Maria Cármen Iglesias (Unidade Local de Saúde Braga, Braga, Portugal); Adriana Coutinho (Unidade Local de Saúde Alentejo Central, Évora, Portugal); Ana Bela Correia, Maria Luísa Gonçalves (Hospital dos SAMS, Lisboa, Portugal); Elzara Aliyeva, Sandra Schäfer, Clara Portugal, Isabel Monge, Luísa Sancho (Unidade Local de Saúde Amadora/Sintra, Amadora, Portugal); José Diogo, Filipa Fortunato, Leonardo Carneiro, José Marta, Isabel Nascimento (Unidade Local de Saúde Almada Seixal, Almada, Portugal); Nadiya Kruptsala, Cláudia Fidalgo, Raquel Diaz, Sónia Ferreira, Inês Cravo Roxo, Elmano Ramalheira (Unidade Local de Saúde Região de Aveiro, Aveiro, Portugal); Isabel Vale, Maria João Tomás, José Miguel Ribeiro (Unidade Local de Saúde Viseu Dão - Lafões, Tondela e Viseu, Portugal); Maria Antónia Read, Valquíria Alves, Margarida Monteiro (Unidade Local de Saúde Matosinhos, Matosinhos, Portugal); Dr. João Faria, Margarida Rodrigues (Unidade Local de Saúde Estuário do Tejo, Vila Franca de Xira, Portugal); Sandra Vieira (Unidade Local de Saúde Alto Minho, Ponte de Lima e Viana do Castelo, Portugal); Maria Favila Menezes, Elsa Calado, Bruno Miguel, L. Nogueira Martins, Maria José Rego de Sousa, Paula Pinto, José Germano de Sousa, Ana Custódio (Centro de Medicina Laboratorial Germano de Sousa Portugal Continental, Portugal); Maria Calle, Mariana Bettencourt Viana, Marvin Oliveira, Hugo Macedo, Svetlana Zhelezovskaya, Isaura Terra (Unidade Local de Saúde Tâmega e Sousa, Amarante e Guilhufe, Portugal); Vitória Rodrigues, Sofia Marques, Joana Selada, Patrícia Pereira, Manuela Azevedo [Laboratório SYNLAB Lisboa (Hospital Beatriz Ângelo, Loures, Portugal; Hospital de Cascais, Cascais, Portugal; Hospitais Lusíadas, Portugal; Hospitais Luz, Portugal)]; Jesuína Duarte, Joana Bernardo, Inês Tapadinhas, Ana Filipa Resende, Andreia Bernardo, Luísa Oliveira, Susana Banza (Unidade Local de Saúde Arrábida, Setúbal, Portugal); Ezequiel Moreira, Carla Ferreira (Unidade Local de Saúde Médio Ave, Santo Tirso e Vila Nova de Famalicão, Famalicão, Portugal); Adília Vicente, Cristina Bragança, Maria Lucas (Unidade Local de Saúde Oeste, Caldas da Rainha, Portugal); Paula Gouveia Pestana, Patrícia Amantegui (Unidade Local de Saúde Cova da Beira, Fundão e Covilhã, Portugal); Cristina Mota Preto, Sara F. Sampaio (Centro de Medicina Laboratorial Germano de Sousa Açores, Ponta Delgada, Portugal); Fátima Vale, Joana Ramos, Rita Gralha (Unidade Local de Saúde da Guarda, Guarda, Portugal); Luís Fialho, Paula Gama, Ana Helena Correia (Unidade Local de Saúde do Médio Tejo, Abrantes, Tomar e Torres Novas, Portugal); Paula Pinto (Unidade Local de Saúde da Lezíria, Santarém, Portugal); Ana Jesus (Unidade Local de Saúde do Arco Ribeirinho, Barreiro e Montijo, Portugal); Marisol Lourinha (Hospital Particular do Algarve, Gambelas, Faro, Portugal).

## Data Availability

The datasets presented in this study can be found in online repositories. The names of the repository/repositories and accession number(s) can be found below: https://doi.org/10.5281/zenodo.13867333, https://www.ebi.ac.uk/ena, PRJNA1000441.

## Footnotes

1
https://github.com/B-UMMI/INNUca

2
https://github.com/tseemann/mlst/releases/tag/v2.19.0

3
https://doi.org/10.5281/zenodo.13867333

4
https://github.com/tseemann/abricate

5
https://github.com/B-UMMI/seq_typing
