# Correction: Two *Streptococcus agalactiae* ST283 invasive infections in Portugal raise the possibility of the emergence of this unusual lineage in Europe

**DOI:** 10.3389/fmicb.2026.1805832

**Published:** 2026-02-26

**Authors:** Elisabete R. Martins, Mykyta Forofontov, José Mota Freitas, José Melo-Cristino, Mário Ramirez, Portuguese Group for the Study of Streptococcal Infections

**Affiliations:** 1 GIMM - Gulbenkian Institute for Molecular Medicine, Lisbon, Portugal; 2 Instituto de Microbiologia, Faculdade de Medicina, Universidade de Lisboa, Lisbon, Portugal; 3 Unidade Local de Saúde do Alto Minho, Viana do Castelo, Portugal

**Keywords:** epidemiological surveillance, group B streptococcus, invasive infections, ST283, foodborne, *Streptococcus agalactiae*, whole-genome sequencing

In the published article, there was an error in the **Discussion**, paragraph 3 regarding the attribution of fluoroquinolone resistance-associated QRDR mutations. The article stated that mutations *gyrA* S81L and *parC* S79Y had been previously reported in ST283 isolates by Ouancharee et al. (2025). However, in that study these mutations were identified in other *Streptococcus agalactiae* lineages and not in ST283 isolates.

The incorrect paragraph reads as follows, “The detection of fluoroquinolone resistance-associated QRDR mutations in our isolates is also noteworthy in the context of ST283 epidemiology. Mutations such as *gyrA* S81L and *parC* S79Y, previously described in resistant human ST283 isolates (Ouancharee et al., 2025) and in enrofloxacin-resistant fish-derived strains (Lukkana et al., 2016), are known to confer high-level fluoroquinolone resistance. Given the widespread use of enrofloxacin in freshwater aquaculture in SEA and the established role of raw freshwater fish consumption in ST283 transmission, the presence of these mutations highlights the potential for cross-sector selection, particularly as fluoroquinolones are rarely used to treat human GBS infections and underscores the importance of sustained genomic surveillance to detect and monitor resistant ST283 lineages”.

The correct paragraph should read as, “The detection of fluoroquinolone resistance-associated QRDR mutations in our isolates is noteworthy in the context of ST283 epidemiology. Substitutions such as *gyrA* S81L and *parC* S79Y are well-established determinants of high-level fluoroquinolone resistance in GBS, having been described in resistant human isolates from multiple lineages (Ouancharee et al., 2025) and in enrofloxacin-resistant fish-derived strains from freshwater aquaculture settings (Lukkana et al., 2016). These mutations are present in our isolates as well as other isolates recovered from humans in SEA and The Netherlands (Figure 1). Given the widespread use of enrofloxacin in freshwater aquaculture in SEA and the established role of raw freshwater fish consumption in ST283 transmission, these findings highlight the potential for cross-sector selection, particularly as fluoroquinolones are rarely used to treat human GBS infections and underscore the importance of sustained genomic surveillance to detect and monitor resistant ST283 lineages.”

The original version of this article has been updated.

